# “Dysfunctions” induced by Roux-en-Y gastric bypass surgery are concomitant with metabolic improvement independent of weight loss

**DOI:** 10.1038/s41421-019-0138-2

**Published:** 2020-01-28

**Authors:** Meiyi Li, Zhiyuan Liu, Bangguo Qian, Weixin Liu, Katsuhisa Horimoto, Jie Xia, Meilong Shi, Bing Wang, Huarong Zhou, Luonan Chen

**Affiliations:** 1 Key Laboratory of Systems Biology, Center for Excellence in Molecular Cell Science, Institute of Biochemistry and Cell Biology, Shanghai Institutes for Biological Sciences, Chinese Academy Sciences, Shanghai, 200031 China; 2 Institute of Fudan-Minhang Academic Health System, Minhang Hospital, Fudan University, Shanghai, 201199 China; 3 Molecular Profiling Research Center for Drug Discovery, National Institute of Advanced Industrial Science and Technology, Tokyo, Japan; 4 Shanghai Ninth People’s Hospital, Shanghai Jiao Tong University School of Medicine, Shanghai, 200011 China; 5 Sherman College of Chiropractic, Boiling Springs, SC 29316 USA; 6 Center for Excellence in Animal Evolution and Genetics, Chinese Academy of Sciences, Kunming, 650223 China; 7 School of Life Science and Technology, ShanghaiTech University, Shanghai, 200031 China; 8 Shanghai Research Center for Brain Science and Brain-Inspired Intelligence, Shanghai, 201210 China

**Keywords:** Mechanisms of disease, Transcriptomics

## Abstract

Metabolic surgery has been increasingly recommended for obese diabetic patients, but questions remain as to its molecular mechanism that leads to improved metabolic parameters independently of weight loss from a network viewpoint. We evaluated the role of the Roux limb (RL) in Roux-en-Y gastric bypass (RYGB) surgery in nonobese diabetic rat models. Improvements in metabolic parameters were greater in the long-RL RYGB group. Transcriptome profiles reveal that amelioration of diabetes state following RYGB differs remarkably from both normal and diabetic states. According to functional analysis, RYGB surgery significantly affected a major gene group, i.e., the newly changed group, which represented diabetes-irrelevant genes abnormally expressed after RYGB. We hypothesize that novel “dysfunctions” carried by this newly changed gene group induced by RYGB rebalance diabetic states and contribute to amelioration of metabolic parameters. An unusual increase in cholesterol (CHOL) biosynthesis in RL enriched by the newly changed group was concomitant with ameliorated metabolic parameters, as demonstrated by measurements of physiological parameters and biodistribution analysis using [^14^C]-labeled glucose. Our findings demonstrate RYGB-induced “dysfunctions” in the newly changed group as a compensatory role contributes to amelioration of diabetes. Rather than attempting to normalize “abnormal” molecules, we suggest a new disease treatment strategy of turning “normal” molecules “abnormal” in order to achieve a new “normal” physiological balance. It further implies a novel strategy for drug discovery, i.e. targeting also on “normal” molecules, which are traditionally ignored in pharmaceutical development.

## Introduction

Diabetes can induce a series of associated complications and increase the risk of several cancers^1,2^, which affects both mortality and patient quality of life. Recently, metabolic surgical treatments have been included as antidiabetic interventions for patients with diabetes and obesity by international diabetes organizations due to their rapid and long-term efficacy in reducing the medication burden and ameliorating diabetes complications (e.g., cardiovascular diseases and nonalcoholic fatty liver disease)^3–5^. Amelioration of metabolic parameters following bariatric/metabolic surgery has been supported by numerous studies on randomized clinical trials and an emerging consensus^3,4^. This concept of “metabolic surgery” is based on observed diabetes remission associated with changes in gut microbiomes^6^ and gut hormones^7,8^, bile acid metabolism^9–11^, and reprogramming of glucose homeostasis^12^. However, despite growing interest in metabolic surgery, questions remain regarding its mechanism leading to improved metabolic parameters independent of weight loss and regarding its effectiveness in nonobese diabetic subjects. The metabolic impact of surgery involves multiple and complicated pathways, and few studies have explored the molecular mechanism of metabolic surgery from the perspective of systems biology.

**Fig. 1:**
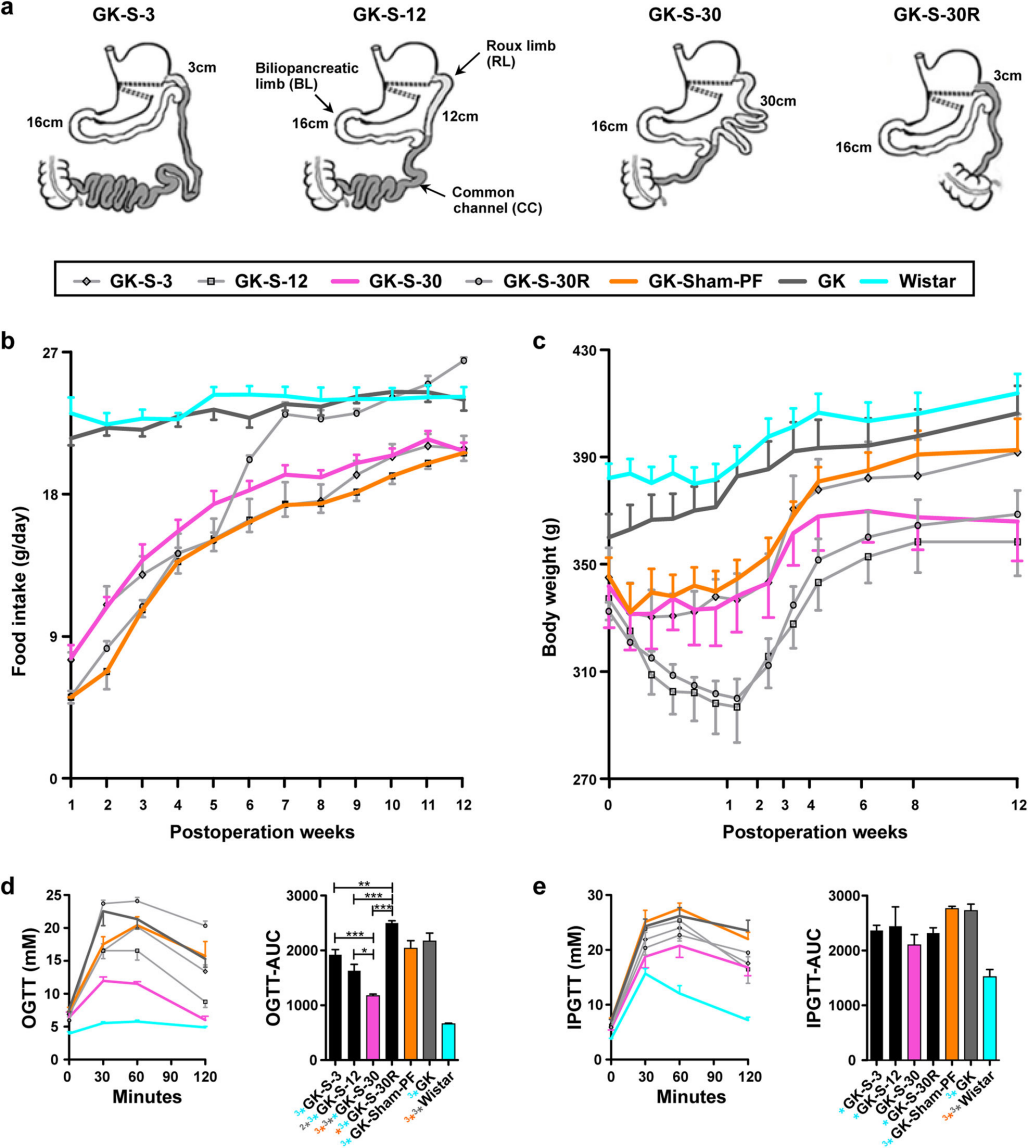
Various RYGB procedures (with regard to RL length) performed in GK rats, and measurement of the corresponding physiological parameters. **a** Schematic description of the various RYGB procedures used in this study. BL, located 16 cm from the ligament of Treitz, was identical in the four surgical groups. RL (light gray), 3, 12, or 30 cm in length, is connected to the upper portion of the stomach and passes food to the CC (dark gray). GK-S-3 group (3-cm RL; very long CC), GK-S-12 group (12-cm RL), GK-S-30 group (30-cm RL; short CC), GK-S-30R group (BL and CC same as in GK-S-30, but 30 cm of RL is excised, and only 3 cm is left). **b**–**e** Physiological parameters in four RYGB groups and three control groups (GK pair-fed/sham-operated (GK-PF), untreated GK (GK), and normal Wistar). Amelioration of diabetes in the seven groups was assessed by measurement of physiological parameters. **b** Food intake. **c** Body weight. **d** OGTT and AUC-glucose. Glucose (2 g/kg) was administered orally. **e** IPGTT and AUC-glucose. Glucose (1 g/kg) was injected i.p. Rats in both **d** and **e** were fasted overnight before experiments. Data are expressed as mean ± SE. AUC was calculated by trapezoidal integration. Statistical significance of differences of means between groups was determined by ANOVA with Tukey’s multiple comparison test (*n* ≥ 5), with *P < 0.05; **(^2^*)*P* < 0.01; ***(^3^*)*P* < 0.001. Symbols with orange color refer to comparison with GK-Sham-PF; gray, with GK; cyan, with Wistar.

Roux-en-Y gastric bypass (RYGB), one of the most effective metabolic operations, excludes a portion of stomach with the proximal intestine (biliopancreatic limb, BL) and rearranges the distal end of the intestine into a Y-configuration, in which food can flow from the upper stomach pouch through the Roux limb (RL) (Fig. 1a)^13^. Studies have demonstrated that RYGB effectively and rapidly improves glycemic control independent of weight loss, and some suggest that changes in metabolic and biological functions in RL in combination with a lack of bile and other digestive fluids play key roles in glucose homeostasis after RYGB^6–15^. One study revealed that glucose uptake was reduced in the RL due to sodium containing bile deprivation after RYGB^9^; others have revealed alterations in distribution and hormone-associated gene expression of enteroendocrine cells compared with those in different intestinal locations, including the BL, RL, and common channel (CC) after RYGB^7^. Saeidi et al. reported that reprogramming, which leads to increased glucose utilization in the RL segment, improves glycemic control following RYGB in diabetic rat models^12^. These findings suggest that the RL plays a role in improving glucose metabolism following RYGB. Lu et al. reported that RYGB in healthy rats and mice reduced insulin production and sensitivity but still maintained cross-tissue glucose homeostasis through alteration of non-insulin determinant metabolic pathways^16^. However, it remains unclear how changes in the RL tract are systematically associated with metabolic improvement for nonobese diabetic subjects independent of weight loss following metabolic surgery. Assessing the functions of gastrointestinal tract segments from the comprehensive biological network viewpoint will not only benefit patients by providing more effective metabolic surgical procedures, but also improve our understanding of the molecular mechanisms of diabetes remission after metabolic surgery, to further guide and extend clinical implementation.

To address the above issues, we designed a series of surgical procedures in nonobese diabetic rat models to assess the relationship between amelioration of metabolic parameters independent of weight loss and RL length in the RYGB procedure, and studied the molecular mechanism of the RL from a systematic and comprehensive viewpoint. Improvements in glucose and lipid parameters were greater in the long-RL RYGB group than in other operated RYGB groups or in Goto-Kakizaki (GK) controls. Diabetes remission by RYGB was found to differ remarkably from both the healthy and diabetic states in analysis of transcriptomic profiles of three rat models representing different physiological states. Functional analysis revealed that RYGB surgery significantly affected one group of genes, i.e., the newly changed group, which showed no differential changes between normal controls (Wistar rats) and GK controls but exhibited significant changes after RYGB surgery. This group of genes, in comparison with those in normal controls, was originally “normal” in GK rats but became “abnormal” in RYGB rats despite diabetes amelioration. We demonstrate here that novel “dysfunctions” of the newly changed group induced by RYGB are involved in amelioration and rebalance metabolism. In particular, the unusual increase in newly synthesized cholesterol (CHOL) enriched by the newly changed group is concomitant with amelioration of metabolic parameters, as demonstrated by measurement of physiological parameters and biodistribution analysis using [^14^C]-labeled glucose. Our findings demonstrate metabolic rebalance by RYGB-induced “dysfunctions” in the newly changed group as a compensatory role contributes to diabetes remission. Rather than attempting to normalize “abnormal” molecules, we suggest a new disease treatment strategy of turning “normal” molecules “abnormal” in order to achieve a new “normal” physiological balance. It further implies a novel strategy for drug discovery, i.e. targeting also on “normal” molecules, which are traditionally ignored in pharmaceutical development.

## Results

### Metabolic improvement is greater in the long-RL (30% of total intestine) group of nonobese GK rats

Significant changes in absorption and metabolism in the RL play roles in diabetes remission by RYGB for obese diabetic subjects^3,9,12^. Accordingly, we evaluated beneficial effects in diabetes remission by RYGB and relationships between such effects and RL length in the surgical procedure for diabetic but nonmorbidly obese subjects. We performed RYGB procedures with various RL lengths in nonobese GK rats (Fig. 1a; see Materials and Methods, “Surgical procedures” for details). In human clinical studies, a normal RL is ~12–15% of total intestinal length, which corresponds to the GK-S-12 group, while a super-long RL is ~24–30% of total intestinal length^17^, which corresponds to that in our GK-S-30 group. Next, we measured several physiological indices related to diabetes (i.e., food intake, body weight, oral glucose tolerance test (OGTT), and intraperitoneal (i.p.) glucose tolerance test (IPGTT)). In comparison with the control groups (i.e., Wistar and GK rats without surgery), the five surgical groups showed remarkably reduced food intake at 72 h postoperation after adapting to normal chow. These differences persisted during weeks (wk) 1–6 postoperation. After wk 6, food intake did not differ significantly between the surgical and control groups. The sham operated surgical control pair-fed GK group (GK-Sham-PF) was given an amount of food equivalent to the smallest amount among the three RYGB groups (see Materials and Methods), usually that of GK-S-12 (Fig. 1b).

Body weight in the five surgical groups declined greatly during wk 1 and then gradually increased, whereas body weight in the two non-surgical control groups showed a steady, gradual increase (Fig. 1c). At 8 wk postoperation, body weight of the GK controls differed significantly from those of the GK-S-12 (regular RL), GK-S-30 (long RL), and GK-S-30R (RL excised) groups, but not from those of the GK-Sham-PF and GK-S-3 (short RL) groups. These findings indicate that the GK-S-12, GK-S-30, and GK-S-30R surgeries, but not the GK-S-3 surgery, were effective in maintaining low body weight. Similarly, a human study showed a positive correlation of RL length with weight loss^18^. OGTTs and IPGTTs were performed 12 wk postoperatively to assess glucose metabolism. In the OGTTs, all six GK groups showed reduced glucose tolerance in comparison with the age-controlled Wistar group (Fig. 1d). Among the five surgical groups, the area under the curve (AUC)-glucose values of the GK-S-12 and GK-S-30 groups were significantly lower than those of the GK group. The AUC-glucose value for GK-S-30 was significantly lower than those of GK-S-12, GK-S-3, and GK-S-30R (Fig. 1d). For IPGTT-AUC values, there were no significant differences in GK-S-30 rats, but remarkable increases in both GK and GK-Sham-PF rats in comparison with Wistar rats (Fig. 1e). These results indicated that nonobese diabetic GK rats benefited from long-RL RYGB surgeries in comparison with other types, and restricted food intake had no positive effects in glycemic controls. In comparison with BL and CC segments, the RL appeared to play a key role in glucose homeostasis following RYGB in nonobese diabetic GK rats; the metabolic benefits disappeared in the GK-S-30R group, which had an identical BL and CC but excised RL in comparison with those in GK-S-30 rats.

### Amelioration of diabetes state following RYGB differs from both normal and diabetic states in RL in terms of gene expression

**Fig. 2:**
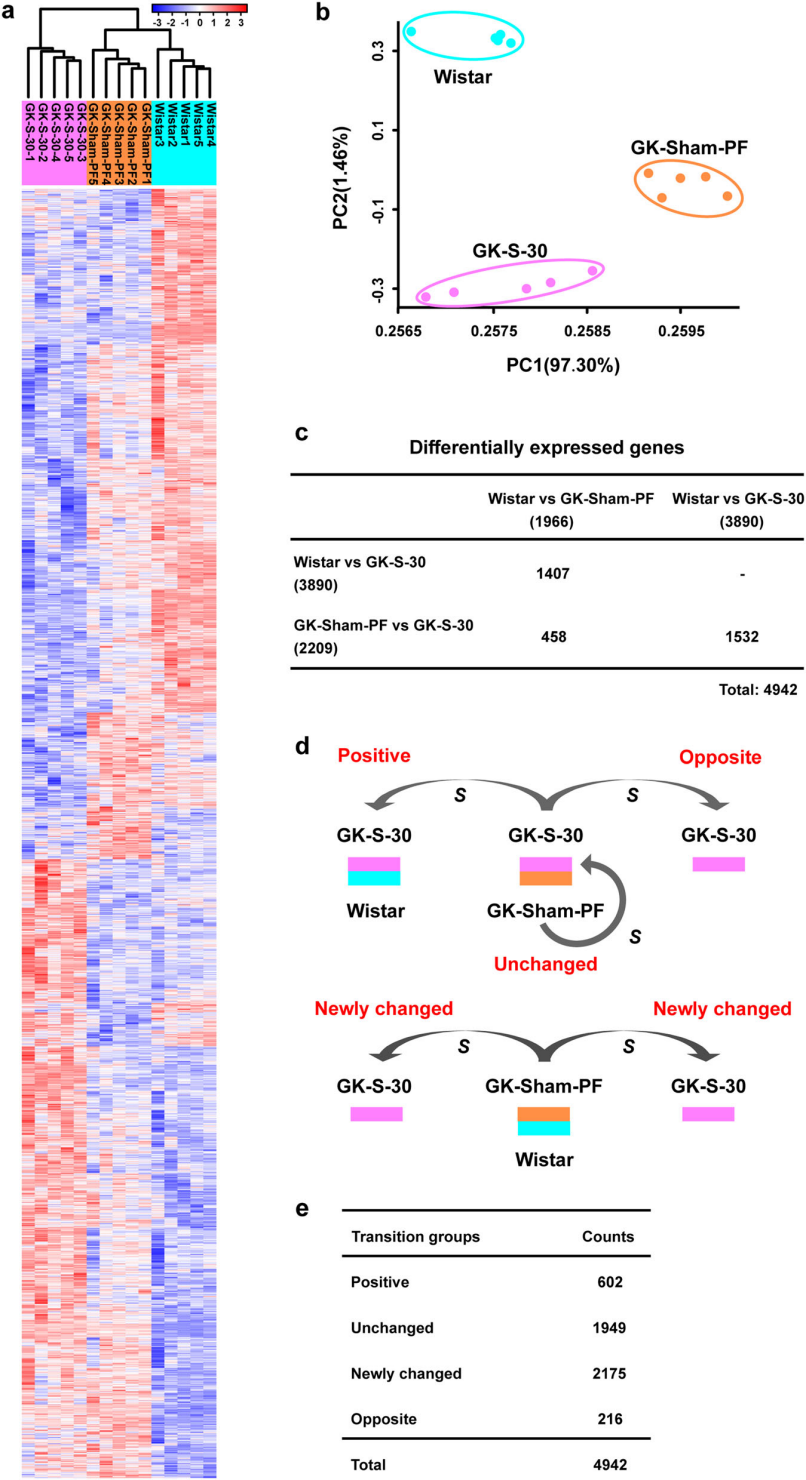
Analyses of transcriptomic profiles among three physiological states. **a** Heatmap illustrating dynamics of 4942 DEGs among the three state groups (normal Wistar, diabetes-GK-PF, diabetes remission-GK-S (short for GK-S-30)). Unsupervised hierarchical clustering was performed to distinguish among physiological states. The three types of rats were distinctly clustered into three independent groups, indicating that physiological state of diabetes remission by RYGB differed strongly from both normal and diabetic states. **b** PCA results showing visually that the three types of rats were clustered distinctly into three independent groups, confirming similar results from hierarchical clustering. **c** Few overlapping DEGs in comparison with pairwise groups, with no statistical significance by the hypergeometric test (all *P* values are 1). **d** Schematic diagram illustrates four transition groups (positive, unchanged, opposite, and newly changed) of DEGs to fractionize functional roles of DEGs and clarify relationships between RYGB-associated and diabetes-associated changes at the gene expression level. S: surgery. **e** DEGs in the four transition groups were counted. Most of the DEGs belonged to the unchanged and newly changed groups. Positive group was out of proportion to unchanged group.

Generally, a whole-genome expression profile is analyzed to characterize a living state as well as to understand the underlying molecular mechanisms to maintain this state^19^. Improvements in glucose metabolism were clearly observed in GK-S-30 but not in GK-S-30R (RL excised) rats. Therefore, we measured whole-genome expression profiles of RL in GK-S-30 rats and the corresponding intestinal segment of GK-Sham-PF and Wistar rats to evaluate molecular changes in the RL resulting from RYGB. We identified 4942 differentially expressed genes (DEGs) by multiple *t*-tests (Fig. 2a and Supplementary Table S1) to characterize three physiological states (i.e., normal, diabetes, and amelioration of diabetes by RYGB). We performed unsupervised hierarchical clustering (Fig. 2a) and principal component analysis (PCA) (Fig. 2b) based on the 4942 identified DEGs. We observed, unexpectedly, that the three groups of rats representing different pathological and physiological states were distinctly clustered into three independent groups, indicating that the state of diabetic amelioration following RYGB was remarkably different from both the normal and diabetic states. Next, we counted overlapping DEGs between two pairwise states and estimated their overlapping significances using a hypergeometric test (Fig. 2c). RYGB-associated DEGs (i.e., GK-S-30 vs GK-Sham-PF rats) had relatively few overlaps with diabetes-associated DEGs (i.e., GK-Sham-PF vs Wistar rats), indicating that RYGB may not recover all molecular “dysfunctions” associated with diabetes but still improves glycemic control.

### DEGs can be categorized into four transition groups for representing different directions of change induced by RYGB

To further clarify the molecular mechanisms of diabetes remission by RYGB, we defined four transition groups (positive, unchanged, opposite, and newly changed groups) (Fig. 2d and Supplementary Table S1) against surgery to fractionize the functional roles of DEGs and interpret relationships with RYGB-associated changes at the gene expression level. The positive group represented diabetes-associated gene expression recovered by RYGB, including DEGs without significant differences between GK-S-30 and Wistar rats, but with significant differences between GK-Sham-PF and Wistar rats or between GK-Sham-PF and GK-S-30 rats. The unchanged group represented diabetes-associated gene expression not improved by RYGB, including DEGs without significant differences between GK-S-30 and GK-Sham-PF rats, but with significant differences between GK-Sham-PF and Wistar rats or between GK-S-30 and Wistar rats. The opposite group represented diabetes-associated gene expression relatively worsened by RYGB, including DEGs with significant differences among GK-Sham-PF, GK-S-30, and Wistar rats. Opposite changing directions of GK-S-30 and Wistar rats were in comparison to corresponding differences between GK-Sham-PF and Wistar rats. The newly changed group represented diabetes-irrelevant genes abnormally expressed after RYGB, including DEGs without significant differences between Wistar and GK-Sham-PF rats, but with significant differences between GK-S-30 and Wistar rats or between GK-S-30 and GK-Sham-PF rats. Thus, gene expression of these newly changed genes in GK-Sham-PF rats was originally at the same level as that in normal (Wistar) rats, but did not remain “normal” after RYGB and actually underwent significant changes to “abnormal” despite amelioration of diabetes.

Next, we individually separated DEGs into these four transition groups. We found, unexpectedly, that most of the DEGs belonged to the unchanged and newly changed groups, while the positive group representing recovered genes was relatively small (Fig. 2e). The big unchanged gene group demonstrated that functional recovery of most diabetes-associated genes is difficult. We therefore hypothesized that newly emerging genes belonging to the newly changed group may play a compensatory roles in amelioration of diabetes following RYGB.

### A rebalance strategy following RYGB: the newly changed group contributes to metabolic rebalance

**Fig. 3:**
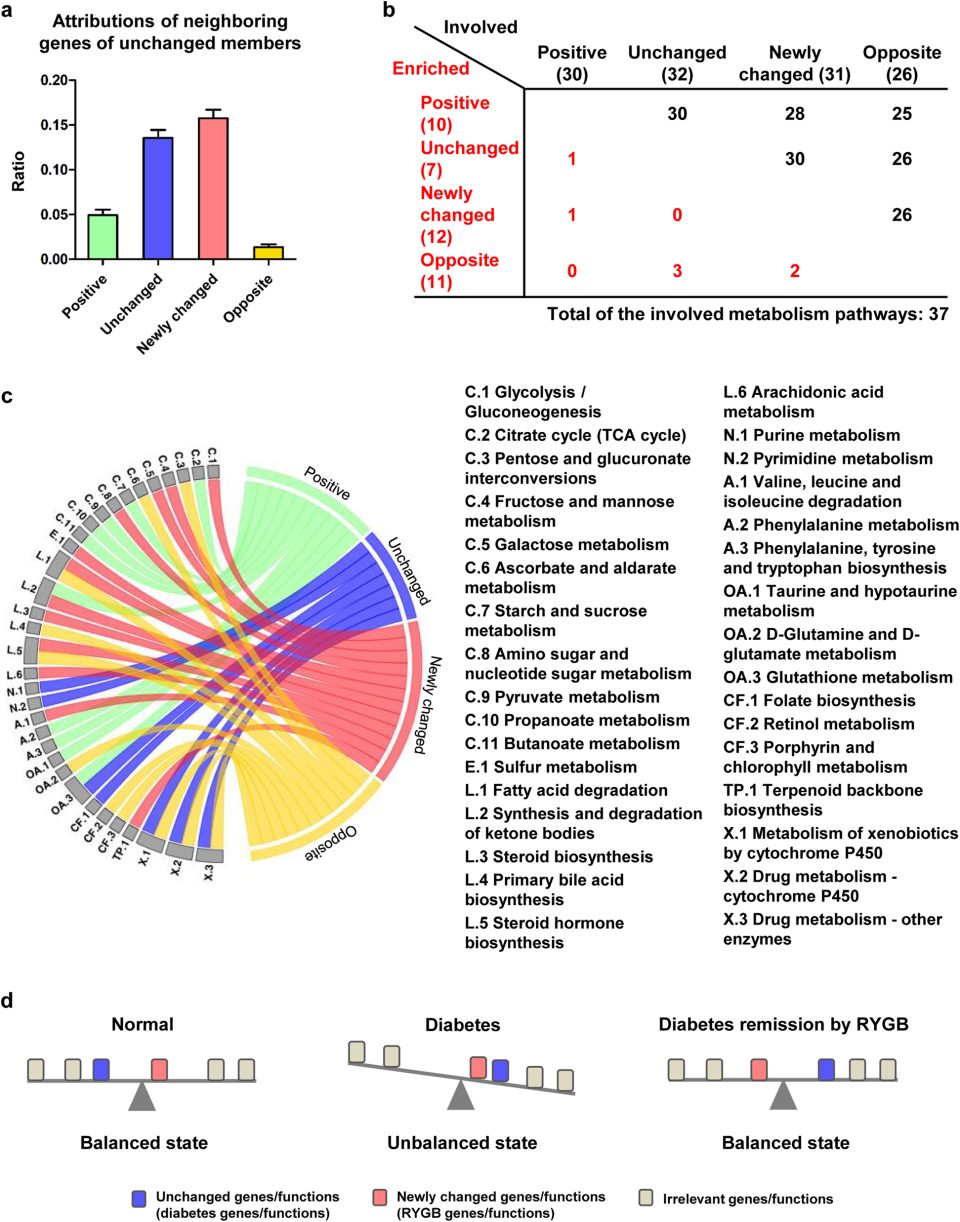
Rebalance model to explain compensatory strategy of diabetes remission by RYGB. **a** Histogram showing distributions of neighboring nodes of unchanged genes in four transition groups. **b** Overlapping functional analysis in a total of 37 metabolism-associated processes significantly enriched by each of four transition groups. **c** Enriched metabolism-associated pathways by four transition groups graphically shown in detail. *C* carbohydrate metabolism, *E* energy metabolism, *L* lipid metabolism, *N* nucleotide metabolism, *A* amino acid metabolism, *OA* metabolism of other amino acids, *CF* metabolism of cofactors and vitamins, *X* xenobiotic biodegradation and metabolism. **d** Schematic diagram illustrating rebalance strategy of diabetes remission by RYGB. Blue block: diabetes-related genes/functions not recovered by RYGB (unchanged group). Red block: newly changed genes, i.e., diabetes-irrelevant genes unusually expressed after RYGB. Not all diabetes-related genes could be restored following RYGB, but the physical system rearranges other normal metabolic pathways, newly changed “dysfunctions”, which unexpectedly improve metabolic parameters and rebalance the abnormalities caused by unchanged diabetes-associated genes. In **a** and **c**, positive transition group is indicated by green color, unchanged group by blue, newly changed group by red, and opposite group by yellow.

Diabetes (particularly type 2 diabetes (T2D)) remission by RYGB has generally been considered to result from functional changes in a complex and systemic interplay of multiple molecules and pathways. We further examined the functional relationships of genes in the four transition groups from molecular interactions and metabolic pathways to more comprehensively understand the underlying mechanisms of diabetes remission by RYGB. After mapping all DEGs into the knowledge-based molecular interactions of rats (Supplementary Fig. S1) and counting individual nodes (i.e., DEGs) neighboring those of the unchanged group, we found that neighboring nodes of the unchanged genes belonged mainly to the newly changed and unchanged groups (Fig. 3a and Supplementary Table S2).

To clarify the functional roles of the newly changed group for amelioration of metabolic parameters by RYGB, our analysis focused on a total of 37 metabolism-associated pathways significantly enriched by at least one of the four transition groups. Although each of the four groups was enriched in its preferred and specific metabolism pathways, there were few overlapping enriched pathways of metabolism among them. Gene members in the four groups could participate or be involved in many other metabolic processes that were enriched by other transition groups, and some of the preferred and specific pathways corresponding to one group had some functional associations (Fig. 3b; Supplementary Fig. S2 and Table S3).

Interestingly, we found after performing functional analyses with members of each transition group (Supplementary Table S3) that the functions enriched by the positive group were involved mainly with carbohydrate metabolism and the tricarboxylic acid (TCA) cycle (Fig. 3c, pathways starting with C), and amino acid metabolism (pathways starting with A or OA). The unchanged group was enriched in nucleic acid and drug metabolism (pathways starting with N and X). The opposite group was more widely spread. Particularly, metabolic pathways involved with carbohydrate metabolism (e.g., glycolysis, gluconeogenesis), amino sugar and nucleotide sugar metabolism, and lipid metabolism (pathways starting with L) were enriched in the newly changed group. These findings support our hypothesis that the newly changed group plays a compensatory role to unchanged dysfunctions in rebalancing glucose homeostasis.

We proposed a rebalance model to explain this strategy of diabetes remission by RYGB (Fig. 3d). Generally, the body's physical system is in equilibrium and balance with organized, synergic, and regulatory metabolic pathways under normal or healthy conditions, whereas if diabetes-associated genes or functions shift and become overwhelmed, the physical system enters an unbalanced state and symptoms of diabetes (e.g., hyperglycemia and hyperlipidemia) occur. After treatment with RYGB, amelioration of diabetes may result from not only functional recovery of diabetes-associated genes but also newly emerging or RYGB-induced “dysfunctions” as a compensatory role for rebalance, as demonstrated in healthy rats and mice after RYGB by the cross-tissue study^16^. In our schematic diagram (Fig. 3d), the blue block is shifted to the right, indicating diabetes-associated genes, which remain unchanged following RYGB. The red block, which previously did not differ between normal and diabetes, is shifted to the left, indicating a newly changed “dysfunction” following RYGB. Different from a traditional recovering of the disease-associated genes, the new strategy of making “normal” functions “abnormal” unexpectedly rebalances and contributes to diabetes remission.

### An unusual increase in newly synthesized CHOL as an example of RYGB-induced “dysfunctions”

Functional analysis indicated that the newly changed genes associated with metabolic pathways were enriched in the carbohydrate and lipid metabolism categories, which may play a compensatory role in rebalancing diabetes parameters. To further map the major RYGB-induced changes in carbohydrate and lipid functional pathways, we obtained signatures of 74 genes that differed (false discovery rate (FDR) <0.05) between GK-S-30 and GK-Sham-PF. Our enrichment analysis revealed nine significant pathways (Supplementary Table S4; FDR <0.05) based on gene sets in MSigDB, of which four were observed in CHOL metabolism. After RYGB, lack of bile may lead to alterations of CHOL uptake and synthesis in the small intestine. Bile has a direct inhibitory effect on cholesterogenesis in the intestinal mucosa^20^. CHOL synthesis may be enhanced in the bile-deprived RL, and this process requires costly glucose consumption to produce very large amounts of basic materials (e.g., acetyl-CoA, ATP, NADPH)^21^, which may have a direct beneficial effect on glycemic control. The intestine, like the liver, is an important site for high-density lipoprotein (HDL) synthesis and secretion and provides ~30% of plasma HDL^6^. We therefore focused on CHOL metabolism enriched by the newly changed group to elucidate its association with metabolic improvement by RYGB.

**Fig. 4:**
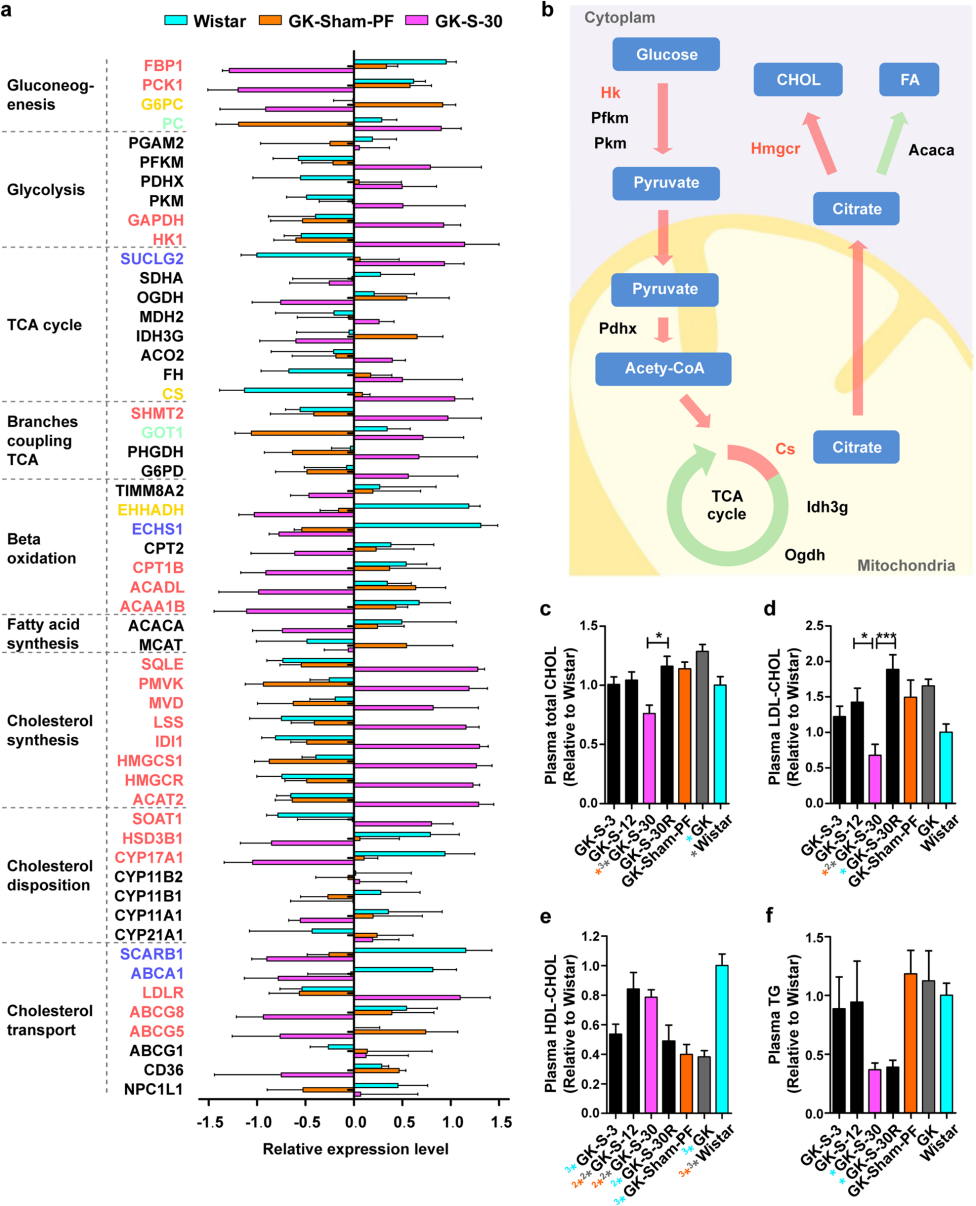
Increase in newly synthesized HDL-CHOL contributes to glycometabolism rebalance. **a** Changes in expression of key enzymes of glucose and CHOL metabolism in three groups (Wistar, GK-PF, GK-S-30; each *n* = 5) in terms of relative mRNA level. Gene symbols are colored green for positive transition group; blue: unchanged; red: newly changed; yellow: opposite; black: non-DEGs. **b** Schematic diagram showing that increased de novo CHOL synthesis in RL has direct beneficial effect on glycemic control after RYGB. **c** Plasma total CHOL, **d** plasma LDL-CHOL, **e** plasma HDL-CHOL, and **f** plasma TG were measured in overnight-fasted blood samples from GK and Wistar groups. Data are expressed as mean ± SE. Statistical terms (*n* ≥ 6) and symbol colors as in Fig. 1.

To further test our hypothesis, we mapped the rate-limiting enzymes involved mainly in glucose, fatty acid, and CHOL metabolism to show their mRNA expression changes following RYGB (Fig. 4a). Red-colored gene symbols, representing the newly changed group, indicate that such gene expression does not differ significantly between GK-Sham-PF and Wistar rats, but is notably activated or inhibited in the RL following RYGB. In glycolysis, most of the key enzymes were upregulated after RYGB. In particular, the rate-limiting enzyme hexokinase (Hk) belonged to the newly changed group. Gluconeogenesis was downregulated (e.g., fructose-1,6-bisphosphatase (Fbp), phosphoenolpyruvate carboxykinase (Pck), and glucose-6-phosphatase (G6pc)). After glycolysis, pyruvate comes into mitochondria and is converted to acetyl-CoA, which enters the TCA cycle. In the TCA cycle, mRNA level is significantly elevated for citrate synthase (CS), but not for isocitrate dehydrogenase (Idh3g) or alpha-ketoglutarate dehydrogenase (Ogdh), indicating that citrate may accumulate and shuttle out of mitochondria to the cytoplasm for either fatty acid or CHOL biosynthesis. Acetyl-CoA carboxylases (Acaca), key enzymes catalyzing the irreversible carboxylation of acetyl-CoA to produce malonyl-CoA for fatty acid synthesis, were not transcriptionally upregulated. These findings suggest that conversion of glucose to CHOL synthesis may become “dysfunctionally” active in RL after RYGB, and contribute to metabolic rebalance (Fig. 4b).

We found that the relative mRNA levels of most enzymes related to CHOL synthesis were significantly increased in comparison with those in both Wistar and GK-Sham-PF rats (new changes) (Fig. 4a). For HMG-CoA reductase (Hmgcr), the rate-limiting enzyme in CHOL synthesis, relative mRNA level in RL was >2-fold higher in GK-S-30 rats than in either Wistar or GK-Sham-PF rats. Intestinal low-density lipoprotein (LDL) receptor (Ldlr) mRNA levels were significantly higher following RYGB, indicating increased internalization of LDL-CHOL from the blood into intestinal cells (Fig. 4a). ABCG5 and ABCG8 at the mRNA level were significantly reduced following RYGB (Fig. 4a), consistent with the concept that RL promotes extracellular CHOL transport across the brush border membrane to prevent excessive biliary CHOL loss^22^ (Fig. 4b).

Precursors (e.g., ATP, acetyl-CoA) of CHOL synthesis may arise from beta-oxidation of fatty acids; however, this mechanism is not supported by mRNA expression of carnitine palmitoyl transferase (Cpt) and acyl-coenzyme A dehydrogenase (Acad) (Fig. 4a). These raw materials are usually derived from glucose (Fig. 4b). Dietary glucose absorbed from their luminal side is strongly reduced in bile-deprived RL^9^; blood glucose taken up from the basolateral side must therefore be increased to meet the requirement for unusually high CHOL biosynthesis in RL.

To validate our hypothesis regarding unusually increased CHOL synthesis (mainly HDL-CHOL) in RL following RYGB, we measured total CHOL, LDL-CHOL, HDL-CHOL, and triglyceride (TG) levels in blood samples from the seven groups after overnight fasting. Total CHOL and LDL-CHOL were much lower in GK-S-30 than in GK-Sham-PF and GK (Fig. 4c, d). HDL-CHOL was much higher in GK-S-30 and GK-S-12 than in GK-Sham-PF and GK (Fig. 4e). LDL-CHOL profiles of GK-S-30R (RL excised) rats were significantly higher (*P* < 0.001) than those of GK-S-30 rats, and were not notably lower than those of GK rats. These findings indicate that RL contributes to HDL-CHOL, which decreases total CHOL and LDL-CHOL levels. In contrast, fatty acid biosynthesis is not increased in RL; TG levels in GK-S-30 and GK-S-30R were similar to each other and significantly lower than those in GK (Fig. 4f). The extremely low TG levels were due to reduced absorption without bile in rats with long RL (GK-S-30) or without RL (GK-S-30R). The greater amelioration of glucose and CHOL profiles in GK-S-30 than in GK-S-30R suggested that metabolic improvement was not associated with reduced TG absorption in RL, but rather with unusually high HDL-CHOL biosynthesis in RL.

### Confirming amelioration of diabetes in STZ rats with long-RL RYGB

**Fig. 5:**
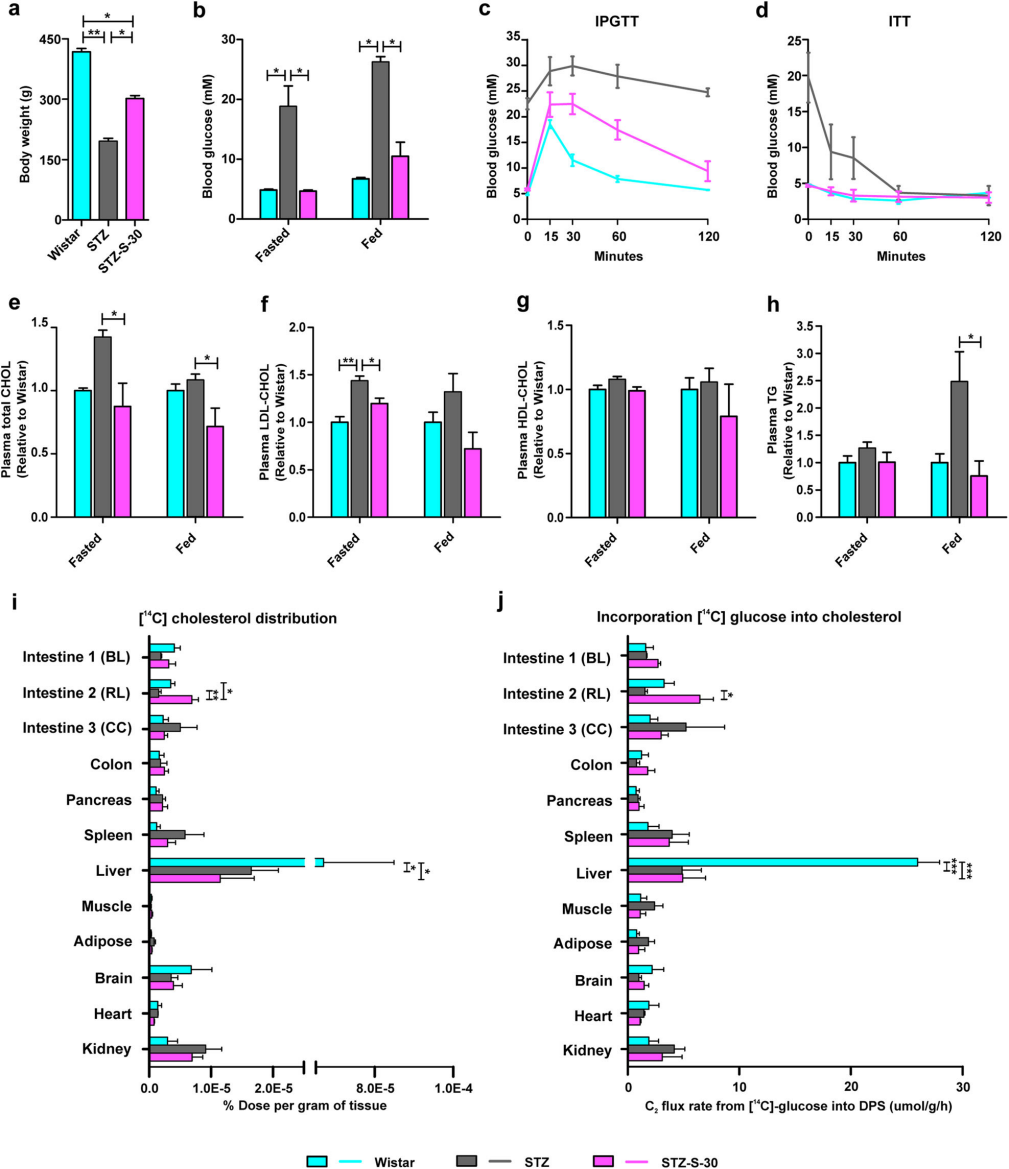
Amelioration of diabetes and changes in [^14^C] glucose metabolism in STZ-treated diabetic rats after long-RL RYGB. **a**–**j** Amelioration of diabetes in 30-cm RL RYGB STZ rats (STZ-S-30) was compared with both nonoperated STZ rats (STZ) and normal Wistar rats by measurement of physiological parameters 3 months postoperation. **a** Body weight. **b** Blood glucose. **c** IPGTT (1 g/kg glucose was injected i.p.). **d** Insulin tolerance test (ITT) (1 U/kg insulin was injected i.p.). **e** Plasma total CHOL. **f** Plasma LDL-CHOL. **g** Plasma HDL-CHOL. **h** Plasma TG. Rats were in fasted-overnight and fed states. **i** [^14^C] glucose was administered orally according to body weight. Three hours later, [^14^C] CHOL was precipitated and biodistribution was measured in the three groups. Newly synthesized CHOL from [^14^C] glucose was significantly increased in intestine 2 (RL) but remained low in liver. **j** [^14^C] glucose incorporation rate into CHOL. Data are expressed as µmol [^14^C] glucose incorporated into digitonin-precipitable sterols (DPS) per gram of tissue per hour. Data are expressed as mean ± SE. Statistically significant differences of means between groups were determined by ANOVA with Tukey’s multiple comparison test (in **a**–**h**, *n* ≥ 4; in **i** and **j**, *n* = 3 for each group), where *P < 0.05, **P < 0.01 for multiple comparisons among the three groups by ANOVA.

We examined the possible beneficial effects of long-RL RYGB on metabolic parameters in another nonobese rat model (streptozotocin (STZ)-treated diabetic rats) to rule out limitations in data interpretation associated with use of a single-animal model. The same RYGB procedure performed in GK-S-30 rats was performed in STZ-treated diabetic rats (STZ-S-30), with nonoperated age-matched Wistar and STZ rats as controls. Three months after RYGB, body weight was significantly higher in STZ-S-30 rats than in STZ controls (Fig. 5a), and glycemic control in the STZ-S-30 rats was greatly improved (Fig. 5b–d). Lipid profiles showed that total CHOL, LDL-CHOL, and TG were significantly reduced in the STZ-S-30 group compared with the STZ group, whereas HDL-CHOL levels did not differ significantly among the Wistar, STZ-S-30, and STZ groups (Fig. 5e–h). To address these findings differently from those in GK rats with surgery, we used oral loading of [^14^C] glucose to confirm the increase in newly synthesized HDL-CHOL labeled by an isotope in STZ-S-30 rats following RYGB (Supplementary Fig. S3). Our results were consistent with the beneficial effects of long-RL RYGB in STZ rats, and confirmed that long RL is associated with amelioration of metabolic parameters.

### Unusually high CHOL biosynthesis in RL by diversion of ingested [^14^C] glucose after RYGB

To further evaluate high CHOL biosynthesis as a new change in RL following RYGB, we analyzed isotope levels in CHOL following oral loading of [^14^C] glucose in long-RL RYGB (STZ-S-30), STZ, and Wistar groups. Three hours after oral loading of [^14^C] glucose, glucose biodistribution per gram (g) tissue was similar in the three groups, i.e., levels were highest in liver, brain, pancreas, and colon, and lowest in adipose tissue and muscle (Supplementary Fig. S4). Glucose uptake was 0.34 ± 0.12% dose per g tissue in RL of RYGB-treated rats vs. 0.17 ± 0.03% in corresponding intestine of STZ-treated rats, which was nearly twice as high but did not achieve significance by ANOVA test. We precipitated and quantified [^14^C] CHOL, which was newly synthesized from orally administered [^14^C] glucose (Fig. 5i). [^14^C] CHOL biodistribution was highest in liver for all three groups, and remained significantly lower in liver after RYGB in comparison with Wistar. Labeled CHOL distribution in intestine 2 did not differ significantly between STZ and Wistar. However, this labeled signal in RL (intestine 2) of STZ-S-30 was almost fourfold higher than that in STZ and twofold higher than that in Wistar (*P* = 0.0057 and 0.0419, respectively; Fig. 5i), and was considered an abnormal change. We calculated the ratio of CHOL biosynthesis to glucose disposal (Fig. 5j). Incorporation of [^14^C] glucose into CHOL was not recovered in liver after RYGB. However, in comparison with STZ, STZ-S-30 showed a significantly upregulated ratio of C_2_ flux into digitonin-precipitable sterols (DPS) in RL. These findings indicate an enhanced rate of glucose incorporation into CHOL synthesis per g tissue per hour in RL. The intestine contributes 30% of HDL-CHOL; we therefore measured newly synthesized plasma HDL-CHOL in RL of STZ-S-30 and STZ using a [^14^C] tracer, and observed a significant increase in newly synthesized plasma HDL-CHOL in STZ-S-30 (Supplementary Fig. S3).

## Discussion

Better understanding the mechanisms of diabetes remission independent of weight loss will facilitate selection of personalized surgical procedures, and development of new, less-invasive treatments for non-morbidly obese patients. In this study, we compared various RYGB procedures with differing RL lengths on metabolic parameters in nonobese diabetic rats. Amelioration of diabetes was greater in the long-RL RYGB group. Following improvement in a disease, we generally expect to see disease-related genes return completely or partially back to normal. However, transcriptome profiles in RL of GK-S-30 group were found to differ remarkably from those in corresponding segments of Wistar and GK-Sham-PF groups. According to functional analysis, RYGB surgery significantly affected a major gene group, i.e., the newly changed group, which was originally “normal” but became “abnormal” in the RYGB group, despite diabetes improvement. We hypothesize that novel “dysfunctions” carried by this newly changed gene group induced by RYGB rebalance diabetic states and contribute to amelioration of metabolic parameters. Unusually increased CHOL biosynthesis enriched by the newly changed group was concomitant with amelioration of metabolic parameters, as demonstrated by measurement of physiological parameters and biodistribution analysis using [^14^C]-labeled glucose. Our findings demonstrate RYGB-induced “dysfunctions” in the newly changed group as a compensatory role contributes to amelioration of diabetes. Rather than attempting to normalize “abnormal” molecules, we suggest a new disease treatment strategy of turning “normal” molecules “abnormal” in order to achieve a new “normal” physiological balance. It further implies a novel strategy for drug discovery, i.e., targeting also on “normal” molecules, which are traditionally ignored in pharmaceutical development.

This study systemically evaluated metabolic parameters in relation to RL length. Improved levels of plasma glucose and CHOL, particularly HDL-CHOL, were most notable in the long-RL RYGB group (GK-S-30) (Figs. 1b–e and 4c–f). In another nonobese rat model (STZ–treated diabetic rats), long-RL RYGB showed similar results (Fig. 5a–h and Supplementary Fig. 3), confirming that long-RL RYGB can be beneficial to diabetic patients independent of weight loss. Our study also included GK-S-30R group, in which RL was excised but BL and common limb lengths were identical to those of GK-S-30 group. Comparison of metabolic parameters between GK-S-30 and GK-S-30R indicated that RL, rather than BL and CC, plays key roles in CHOL homeostasis and may contribute to glycemic control following RYGB (Figs. 1b–e and 4c–f). Results for GK-S-30R control suggest that metabolic improvement is due to reprogrammed metabolism rather than to reduced absorption in RL. Gastrointestinal tract changes per se alter metabolism in both RL and whole body, providing physiological and quantitative observations for guiding RYGB procedure in diabetic patients. A clinical trial involving comparison of standard vs. distal RYGB revealed reduction in total and LDL CHOL, fasting glucose, and hemoglobin A1c levels, and elevation in HDL-CHOL levels after distal gastric bypass. Nutritional adverse effects, such as anemia, vitamin deficiency, secondary hyperparathyroidism, and loose stools, were more frequent after distal gastric bypass, although mean weight loss did not differ^23^. We propose long-RL RYGB for diabetic patients without morbid obesity; however, complete consensus guidelines with preoperative evaluations are necessary to avoid severe complications post-operation, and continued development of novel, less-invasive treatments is highly desirable.

Metabolic improvements following bariatric surgery in morbidly obese patients are always accompanied by weight loss and reduced food intake. It is therefore difficult to separate the contributions of these factors vs. the surgery per se. We observed greater improvement in glucose metabolism and lipid profiles in GK-S-30 relative to GK-S-30R rats, although weight loss was equivalent or greater in GK-S-30R, particularly in the beginning (Fig. 1c). GK rats provide a non-obese spontaneous type 2 diabetic animal model with gradually occurring hyperglycemia^24^. Weight loss following RYGB in GK rats has been repeatedly documented. In another non-obese STZ model in the present study, high-dose (50 mg/kg) STZ injection destroyed most pancreatic beta cells immediately, and hyperglycemia occurred the day after injection. Insulin is the major anabolic hormone. Because of greatly reduced beta cell number and presumably decreased insulin levels, body weights of STZ rats did not increase throughout the experiment. Weight gain of STZ rats after RYGB was consistent with results of previous studies^25^ and reflects amelioration of diabetes. The mechanism of weight gain in STZ rats undergoing RYGB may be due to increased pancreatic beta cell area^26^, reduced beta cell apoptosis^27^, increased beta cell proliferation^28^, and/or improved beta cell function^29^, as documented previously in rodents undergoing RYGB. The seemingly contradictory body weight changes in GK and STZ rats following RYGB strongly indicate weight-independent metabolic improvement after RYGB. Changes in food intake cannot account for the beneficial effects of RYGB, since we observed greater metabolic improvement in GK-S-30 despite the fact that food intake was equivalent in GK-S-3 and GK-Sham-PF (Fig. 1b). A sudden increase in food intake was observed in GK-S-30R (the group that has short bowel because of 30-cm small bowel resection) 6 wk after operation. Nutritional adaptations have been reported following small bowel resection in rats, including increased food intake, hyperplasia of remaining bowel remnant, and reduced gastric-inhibitory peptide secretion^30,31^. The sudden increase in our study in food intake in GK-S-30R after 6 wk may reflect an adaptation response to small bowel resection.

After examining transcriptomic profiles by systemic biology analysis, we found unexpectedly that physiological state of diabetes amelioration following RYGB differed remarkably from both normal and diabetic states (Fig. 2a, b). The majority of DEGs belonged to a newly changed group, which originally had expression levels that were similar in diabetes vs. normal, but underwent significant changes to “abnormal” despite diabetes remission. We therefore hypothesized that functional recovery of diabetes-associated genes is sometimes difficult, and newly emerging “dysfunctions” are induced in the body to play a compensatory role for rebalance and maintenance of a new improved state. Based on results of functional analysis (Fig. 3c and Supplementary Tables S3 and S4), we focused on glucose-associated CHOL metabolism to further evaluate the occurrence of “dysfunction” following RYGB and to test our rebalance model. Findings for both mRNA levels (Fig. 4a) and protein levels (Figs. 4c–f and 5i, j) indicated that increased de novo CHOL synthesis in RL was a new change. [^14^C] CHOL in RL of STZ-S-30 rats was nearly four- and twofold higher than in STZ and Wistar rats (Fig. 5i). C_2_ flux rate incorporated into DPS in intestine 2 did not differ significantly between STZ and Wistar, but was significantly higher in STZ-S-30 than in STZ (Fig. 5j). These normalized C_2_ flux rates include corrections for loss of [^14^C] during flow of carbon atoms through CHOL biosynthesis, but do not take into account dilution of acetyl-CoA from unlabeled precursors, such as glycogen and endogenously produced glucose. Bradley reported that suppression of endogenous glucose production in response to oral glucose ingestion returned to baseline level more rapidly following RYGB surgery (1.5 vs. 4 h)^29^. The rate of CHOL synthesis from [^14^C] glucose is, therefore, underestimated to a greater degree in STZ-S-30. Enhanced rate of glucose incorporation into CHOL in RL reflects a diversion of glucose to CHOL synthesis pathway, which may have a direct beneficial effect on glycemic control. These observations support our hypothesis that “normal” CHOL biosynthesis from glucose becomes “abnormally” high in RL following RYGB. Although plasma HDL-CHOL levels did not differ significantly between STZ and STZ-S-30 (Fig. 5g), we confirmed instantaneous increase of CHOL and HDL-CHOL biosynthesis from isotope-labeled glucose in RL in this model (Fig. 5i–j and Supplementary Fig. S3). HDL-CHOL is synthesized not only in small intestine but also in liver; the latter is the major source of plasma HDL-CHOL. Abnormal CHOL biosynthesis in RL had a direct improving effect on metabolic parameters, particularly lipid profiles, after RYGB (Fig. 4c–f for GK rat model and Fig. 5a–h and Supplementary Fig. S3 for STZ rat model), which extends our knowledge of the “midgut”. In the GK-S-30R group (lacking RL), bad CHOL (total-CHOL and LDL-CHOL) levels were strongly increased whereas HDL-CHOL was greatly reduced (Fig. 4c–e).

Can abnormally high CHOL biosynthesis in RL increase glucose uptake from plasma for glycemic control? Glucose sequestered in intestinal segments arises from two dynamic routes: (i) dietary glucose is absorbed from luminal side into intestine and then transferred to blood; (ii) blood glucose is taken up from basolateral side and used for intestinal metabolism. Baud reported that oral glucose absorption in RL was strongly reduced because of diminished sodium concentration^9^. To obtain glucose for enhanced CHOL biosynthesis, which requires costly glucose consumption to produce very large amounts of basic materials (e.g., acetyl-CoA, ATP, NADPH)^21^, blood glucose uptake by RL must be significantly increased to compensate for reduced glucose absorption from luminal side following RYGB surgery. In our study, glucose disposal in intestine 2 (i.e., RL) was higher (but not significantly so) following RYGB (Supplementary Fig. S4). In another study, when [^18^F]-fluorodeoxyglucose (FDG) was administered intravenously, in vivo PET/CT scanning revealed that RL displayed intense plasma FDG uptake 60 min after injection, and became a major tissue site for glucose disposal^12^. The reason for unchanged glucose distribution in our study is that samples were collected 3 h after oral [^14^C] glucose administration, which is a good time for study of newly synthesized CHOL from [^14^C] glucose^32^, but not for analysis of glucose distribution. After RYGB operation, there is almost no retardation in stomach pouch without pyloric sphincter. [^14^C] glucose reaches RL and CC within a few minutes, blood radioactivity returns to basal level 2 h after [^14^C] glucose consumption, and production of unlabeled endogenous glucose also returns rapidly to normal^29,32^. At the time tissues were collected in our study, most [^14^C] glucose had been incorporated into [^14^C] CO_2_ or sequestered by tissues for their own metabolism (e.g., fatty acid or CHOL biosynthesis). Therefore, despite the fact that glucose distribution remained unchanged in RL, level of glucose taken up from blood may have been significantly higher, contributing to improved glucose homeostasis after surgery (Fig. 5c).

It would be very interesting to evaluate the possibility that inhibition of proximal intestinal CHOL biosynthesis counteracts the beneficial effect of surgery on glucose homeostasis, and thereby assess the importance of this new change in amelioration of hyperglycemia. However, no drug (or corresponding mouse model) is currently available that inhibits CHOL biosynthesis only in the small intestine. Liver is the major organ for de novo CHOL biosynthesis, and controls total-CHOL, VLDL-CHOL, and LDL-CHOL levels in blood. Small intestine contributes to blood HDL-CHOL levels^33^. Application of statin drugs to inhibit whole-body CHOL biosynthesis has a greater effect on liver than on small intestine. In a study of statin therapy in gastric bypass patients, statins reduced total CHOL levels (mainly by inhibiting CHOL biosynthesis in liver) and increased HDL-CHOL levels (no inhibition of CHOL biosynthesis in small intestine). The authors concluded that use of statins with gastric bypass may be beneficial to patients by decreasing long-term cardiac risk^34^. Systemic drug screening studies are currently underway in our lab to find chemical reagents that specifically upregulate or downregulate CHOL biosynthesis in small intestine. Initial results indicate that such reagents are strongly involved in bile secretion. Such reagents may be developed as drugs to mimic or inhibit biomolecule changes following RYGB.

Our findings provide novel insights into the molecular mechanism of diabetes remission following RYGB, from the perspective of systems biology; i.e., metabolic rebalance by making “normal” functions “abnormal” in the newly changed group. We found 2175 newly changed DEGs in RL. Of these, 935 are marked in KEGG, and 230 genes were mapped into metabolic pathways (Supplementary Table S3). Among these pathways, we picked cholesterogenesis as one of the many examples of “dysfunctions” induced by RYGB, and provided support for our rebalance model by showing that this new change has an improving effect on lipid profiles and glycemic control in nonobese diabetic rat models. Our findings do not rule out possible contributions by other weight-independent mechanisms. Such potential mechanisms include RL hyperplasia^35^, Glut 1 re-expression in RL^12^, increased fecal calorie output^36^, and alteration of metabolic profile independent of insulin secretion induced predominantly by rearrangement of gastrointestinal tract^16^. Many such mechanisms represent "abnormal" changes, consistent with wide occurrence of “dysfunctional” pathways in RYGB. Through deconstruction and better understanding of the rebalance model, we can target not only disease relevant gene but also generally ignored “normal” biomolecules to develop less-invasive therapies for obesity and diabetes.

## Materials and methods

### Animals

Male 14-wk-old nonobese spontaneous diabetic GK rats and Wistar rats of a Japanese colony were purchased from the National Rodent Laboratory Animal Center (Shanghai, China) and maintained under constant ambient temperature and humidity with a 12-h light/dark cycle and with free ad lib assess to water and standard laboratory chow unless otherwise specified. Three age-matched groups were used as controls: untreated GK (GK), sham-operated and pair-fed GK (GK-Sham-PF), and normal controls (Wistar). For STZ treatment, Wistar rats were injected i.p. with 50 mg/kg STZ (Sigma-Aldrich; St. Louis, MO, USA) dissolved in citrate buffer (0.1 M sodium citrate, pH 4.5). Only rats with blood glucose levels >300 mg/dl the day after injection were studied further. The rate of success in modeling was ~70 percent. Age-matched normal male Wistar and untreated STZ rats were used as controls. Using two different models help to specifically reduce the evoked weight loss effects. All experiments were in compliance with NIH guidelines and the Guide for the Care and Use of Laboratory Animals and were approved by the Chinese Academy of Science Institutional Animal Care and Use Committee.

### Surgical procedures

In this study, the total length of the small intestine from the upper jejunum to the ileocecal valve in adult rats was ~100 cm, as evaluated by peer rats, in agreement with a previous report^37^. A BL was formed as the first section of the intestine from the lower portion of the stomach to a point 16 cm distal to the ligament of Treitz^24^. The end of this limb was joined at various sites of the intestine and rearranged into a Y-configuration to create various RL lengths. The RL was connected to the upper portion of the stomach and passed food to a CC. The different RYGB procedures were 3 cm RL in length for the GK-S-3 group, 12 cm for the GK-S-12 group, and 30 cm for the GK-S-30 group (Fig. 1a). In comparison with rats in the GK-S-30 group, rats in the GK-S-30R group had an equivalent BL, side-to-side jejunoileal anastomosis, and CC, but 27 cm of the 30-cm-RL was excised to leave only 3 cm.

Operations were performed on 16-wk-old GK rats after overnight fasting and under continuous isoflurane anesthesia. The abdomen was opened by a midline incision, and the stomach was mobilized by opening the lesser sac through the greater omentum. The body of the stomach was divided into two pouches between the glandular portion and gastric fundus. The vagus nerve and accompanying right and left gastric vasculature were gently dissected from the lesser curve to avoid damage. A BL extending 16 cm from the ligament of Treitz was transected. The proximal segment was drained into various sites on the small intestine, depending on the surgical group, using a side-to-side anastomosis. The distal segment was anastomosed to the gastric remnant. For the GK-S-30R RL excised group, a 27-cm RL was first excised following ligation of corresponding mesenteric vessels and then connected to the gastric remnant. The abdominal incision was closed by straticulate saturation. For sham-operated animals, following laparotomy, an incision was made at a site 16 cm distant from the ligament of Treitz and then reconnected by side-to-side anastomosis without intestinal rearrangement. The intestine was gently handled for another 30 min before closure of the incision.

After operations, animals were allowed to recover in a warm box and then returned to the animal facility, where they were administered buprenorphine and ampicillin for 3 days. Animals in the surgical groups and GK-Sham-PF group were given only purified water for 12 h following surgery. This provision was followed by a liquid diet containing 5% glucose and 0.2% KCl for the next 48 h and then standard laboratory chow until the end of the study.

Surgical techniques and recovery procedure for STZ-S-30 rats were the same as those for GK-S-30 rats as described above.

### Food intake and body weight monitoring

Preweighed pellets were placed in the cage, and recovered food was weighed the next day to calculate the daily food consumption. The average amount of consumed food for each surgical group was calculated, and an amount equal to the lowest of these values was given to the GK-Sham-PF group. The above procedure was repeated once per wk for the surgical groups, and the food amount for the GK-Sham-PF group was adjusted as needed.

Body weight was measured 1 day before surgery. After surgery, body weight was measured every day for 7 days and once per wk thereafter.

### OGTT and IPGTT

These tests were performed at 11 and 12 wk postoperation in 16-h fasted rats. For OGTT, 2 g/kg glucose was administered orally. For IPGTT, 1 g/kg glucose was injected into the abdomen. Blood glucose levels in tail vein blood were measured at 0, 30, 60, and 120 min. AUC was calculated to estimate glucose tolerance.

### Whole-genome expression profiles

Fourteen wk postoperation, animals were fasted overnight and sacrificed in the morning (between 9 and 11 a.m.). A 5-cm RL segment was cut 5 cm away from the intestinal gastric anastomosis in the GK-S-30 group. The corresponding segments were 21–26 cm from the ligament of Treitz for the Wistar and GK-Sham-PF groups without intestinal rearrangement. For each group, five samples were frozen immediately in liquid nitrogen and stored at −80 °C until microarray analysis.

To assess whole-genome expression, we individually extracted total RNAs from RL samples using TRIZOL Reagent (Life Technologies) and checked for an RIN number to inspect the RNA integrity by an Agilent Bioanalyzer 2100 (Agilent Technologies). The qualified total RNAs were further purified using an RNeasy Mini Kit (QIAGEN) and RNase-Free DNase Set (QIAGEN). Then, total RNAs were amplified and labeled with a One-Color Low Input Quick Amp Labeling Kit (Agilent) according to the manufacturer’s instructions. Array hybridization and washing were performed with constant rotation on the whole rat genome gene expression slide (Agilent). Slides were scanned by a microarray scanner (Agilent) with default settings. Data were extracted using the Feature Extraction software program 10.7 (Agilent), and raw data were normalized using the Quantile algorithm, Gene Spring software program 11.0 (Agilent). All microarray data were deposited into the Gene Expression Omnibus database (http://www.ncbi.nlm.nih.gov/geo/) with accession number GSE126835.

To identify DEGs, we compared gene expression intensities among samples at two different time points using analysis of variance (ANOVA) with Tukey’s multiple comparison test (two-tailed *P* value < 0.05).

### Assays

Glucose was measured by a glucometer (Accu-check, Roche; Indianapolis, IN, USA). Blood samples were kept on ice until plasma preparation (centrifugation at 3000 rpm for 15 min at 4 °C). Plasma was immediately separated and stored at −80 °C until analysis. TG, total-CHOL, HDL-CHOL, and LDL-CHOL from plasma and intestinal supernatant were measured using Chemix-180 (Sysmex Corp.; Mundelein, IL, USA).

### Measurement of [^14^C] glucose biodistribution in vivo

Biodistribution of [^14^C]-labeled glucose in vivo was determined as described previously^38^. Rats were fasted overnight and then gavaged with 250 μCi/kg [^14^C] glucose (New England Nuclear; Boston, MA, USA). After 3 h, liver, spleen, colon, whole blood, pancreas, brain, kidney, heart, brain, adipose, and muscle tissues were cut and weighed. An interval of 3 h was chosen based on a previous study^39^. In the STZ-S-30 group, the intestine was divided into three parts (BL, RL, and CC), which were collected separately and termed intestines 1, 2, and 3, respectively. In the diabetic control group, the intestine was cut into three parts corresponding to BL, RL, and CC. The intestine was washed twice with PBS, and all other tissues were directly minced and saponified according to tissue weights. To determine [^14^C] glucose biodistribution in vivo, 200 μl of saponified samples of all tissues and plasma were collected, and [^14^C] content was determined by liquid scintillation counting.

### Measurement of newly synthesized CHOL from [^14^C] glucose in vivo

This procedure was as described previously^20^ with some modification. Samples were saponified by adding 2 ml of 2.5 M KOH and 2 ml of anhydrous ethanol per g tissue or per milliliter of plasma and heating at 75 °C for 3 h. Samples were then extracted with petroleum ether to remove unsaponifiable lipids. CHOL was precipitated as digitonides by adding 200 μl of 2% digitonin per g tissue or per ml plasma and standing overnight. Samples were centrifuged at 4500 rpm for 30 min, and DPS were washed successively with water, acetone, and diethyl ether. DPS were quantitated using a color development with ferric chloride. Scintillation solution was added to each vial according to the weight, [^14^C] content was measured by scintillation counting, and ratio of glucose incorporated into CHOL was calculated. Data were expressed as µmoles of radioactive glucose incorporated into DPS per g tissue per h. Incorporation rate was calculated by dividing dpm of [^14^C] in DPS by specific activity of [^14^C] glucose in the tissue. To correct for loss of 33% of radioactivity as CO_2_ during sterol biosynthesis, [^14^C] glucose incorporation rates were multiplied by a factor of 1.5. A second correction term was introduced into the calculations in order to express incorporation rates as µmol C_2_ units (i.e., acetyl-CoA into DPS); rates of incorporation were multiplied by a factor of 1.2 (ref. ^40^).

Newly synthesized HDL-CHOL was assayed as previously described^41^. In brief, 50 μl of 4% sodium phosphotungstate and 12.5 μl of 2 M MgCl_2_ were added to 500 μl of plasma. Precipitation of LDL and very low-density lipoprotein (VLDL) occurred immediately. Precipitate was removed by centrifugation (10 min, 6000×*g*), and remaining CHOL in solution was regarded as HDL-CHOL. CHOL in solution was precipitated and measured by scintillation counting of [^14^C] content as above.

### Four transition groups of DEGs

To evaluate potential RYGB/diabetes-associated biological functions affected by the identified genes, we mapped DEGs into four transition groups, i.e., positive, unchanged, newly changed, and opposite groups against surgery, as shown in Fig. 2d. The positive group included genes without significant differences between GK-S-30 and Wistar but with significant differences between GK-Sham-PF and Wistar or between GK-Sham-PF and GK-S-30. The unchanged group included genes without significant differences between GK-S-30 and GK-Sham-PF but with significant differences between GK-Sham-PF and Wistar or between GK-S-30 and Wistar. The opposite group included genes with significant differences among GK-PF, GKS, and Wistar, while opposite changing directions in GKS and Wistar were in reference to those in GK-PF. The newly changed group included genes without significant differences between Wistar and GK-Sham-PF but with significant differences between GK-S-30 and Wistar or between GK-S-30 and GK-Sham-PF. The four transition groups were defined in comparison with surgery.

### Functional analysis

The Kyoto Encyclopedia of Genes and Genomes (KEGG)^42^ was used for canonical pathway detection and functional annotations of genes of the four transition groups. We then estimated enrichment significance of specific proteins in each biological process or pathway based on a hypergeometric test. Significantly enriched functions were chosen on the basis of corresponding *P* value < 0.05 after FDR correction.

Enrichment analysis was performed as described previously^24^. In brief, signatures were characterized using known gene sets with biological functions. We adopted the gene sets in CP (canonical pathways from KEGG, BICARTA, and Reactome) of C2 (curated gene sets) in MSigDB, which contained 871 gene sets that were manually curated from previous reports. We then estimated enrichment probability of genes in the expression signature for each gene set based on hypergeometric probability. If the gene set is composed of *k* genes and *l* genes are detected in the period-specific signature, the probability is calculated as:

$$p\left( {X \le l} \right) = 1 - \mathop {\sum}\limits_{i = o}^1 {\frac{{\left( {\frac{M}{I}} \right)\left( {\frac{{N - M}}{{k - i}}} \right)}}{{\frac{N}{k}}}},$$

where *M* and *N* are the total numbers of genes in the period-specific signature and in the gene set, respectively. The significance probability was set to 0.05.

## Supplementary information

Supplementary Material

Supplementary Table S1

Supplementary Table S2

Supplementary Table S3

Supplementary Table S4

## Supplementary information

**Supplementary Information** accompanies the paper at (10.1038/s41421-019-0138-2).

## References

[1] Giovannucci E (2010). Diabetes and cancer: a consensus report. Diabetes Care.

[2] Pearson-Stuttard J (2018). Worldwide burden of cancer attributable to diabetes and high body-mass index: a comparative risk assessment. Lancet Diabetes Endocrinol..

[3] Rubino F (2016). Metabolic surgery in the treatment algorithm for type 2 diabetes: a joint statement by International Diabetes Organizations. Diabetes Care.

[4] Brito JP, Montori VM, Davis AM (2017). Metabolic surgery in the treatment algorithm for type 2 diabetes: a joint statement by International Diabetes Organizations. JAMA.

[5] Caiazzo R (2014). Roux-en-Y gastric bypass versus adjustable gastric banding to reduce nonalcoholic fatty liver disease: a 5-year controlled longitudinal study. Ann. Surg..

[6] Liou AP (2013). Conserved shifts in the gut microbiota due to gastric bypass reduce host weight and adiposity. Sci. Transl. Med..

[7] Rhee NA (2015). Effect of Roux-en-Y gastric bypass on the distribution and hormone expression of small-intestinal enteroendocrine cells in obese patients with type 2 diabetes. Diabetologia.

[8] Madsbad S, Holst JJ (2014). GLP-1 as a mediator in the remission of type 2 diabetes after gastric bypass and sleeve gastrectomy surgery. Diabetes.

[9] Baud G (2016). Bile diversion in Roux-en-Y gastric bypass modulates sodium-dependent glucose intestinal uptake. Cell Metab..

[10] Kuipers F, Groen AK (2014). FXR: the key to benefits in bariatric surgery?. Nat. Med..

[11] Patti ME (2009). Serum bile acids are higher in humans with prior gastric bypass: potential contribution to improved glucose and lipid metabolism. Obesity (Silver Spring).

[12] Saeidi N (2013). Reprogramming of intestinal glucose metabolism and glycemic control in rats after gastric bypass. Science.

[13] Cohen R, Pinheiro JS, Correa JL, Schiavon CA (2006). Laparoscopic Roux-en-Y gastric bypass for BMI < 35 kg/m(2): a tailored approach. Surg. Obes. Relat. Dis..

[14] Li QR (2018). Systems signatures reveal unique remission-path of type 2 diabetes following Roux-en-Y gastric bypass surgery. EBioMedicine.

[15] Laferrere B (2008). Effect of weight loss by gastric bypass surgery versus hypocaloric diet on glucose and incretin levels in patients with type 2 diabetes. J. Clin. Endocrinol. Metab..

[16] Lu Z (2018). Non-insulin determinant pathways maintain glucose homeostasis upon metabolic surgery. Cell Discov..

[17] Stefanidis D, Kuwada TS, Gersin KS (2011). The importance of the length of the limbs for gastric bypass patients-an evidence-based review. Obes. Surg..

[18] Brolin RE (2005). Long limb Roux en Y gastric bypass revisited. Surg. Clin. North Am..

[19] Li M, Zeng T, Liu R, Chen L (2014). Detecting tissue-specific early warning signals for complex diseases based on dynamical network biomarkers: study of type 2 diabetes by cross-tissue analysis. Brief. Bioinform..

[20] Dietschy JM, Siperstein MD (1965). Cholesterol synthesis by the gastrointestinal tract: localization and mechanisms of control. J. Clin. Invest..

[21] Bloch K (1965). The biological synthesis of cholesterol. Science.

[22] Yu L (2002). Overexpression of ABCG5 and ABCG8 promotes biliary cholesterol secretion and reduces fractional absorption of dietary cholesterol. J. Clin. Invest..

[23] Risstad H (2016). Standard vs distal Roux-en-Y gastric bypass in patients with body mass index 50 to 60: a double-blind, randomized clinical trial. JAMA Surg..

[24] Zhang R (2013). Association of Rev-erbalpha in adipose tissues with type 2 diabetes mellitus amelioration after gastric bypass surgery in Goto-Kakizaki rats. Am. J. Physiol. Regul. Integr. Comp. Physiol..

[25] Shuang J (2015). Relief of diabetes by duodenal-jejunal bypass sleeve implantation in the high-fat diet and streptozotocin-induced diabetic rat model is associated with an increase in GLP-1 levels and the number of GLP-1-positive cells. Exp. Ther. Med..

[26] Speck M, Cho YM, Asadi A, Rubino F, Kieffer TJ (2011). Duodenal-jejunal bypass protects GK rats from {beta}-cell loss and aggravation of hyperglycemia and increases enteroendocrine cells coexpressing GIP and GLP-1. Am. J. Physiol. Endocrinol. Metab..

[27] Chai F (2011). Adiponectin downregulates hyperglycemia and reduces pancreatic islet apoptosis after roux-en-y gastric bypass surgery. Obes. Surg..

[28] Li Z (2010). Roux-en-Y gastric bypass promotes expression of PDX-1 and regeneration of beta-cells in Goto-Kakizaki rats. World J. Gastroenterol..

[29] Bradley D (2012). Gastric bypass and banding equally improve insulin sensitivity and beta cell function. J. Clin. Invest..

[30] Young EA, Weser E (1974). Nutritional adaptation after small bowel resection in rats. J. Nutr.

[31] Yanala UR, Reidelberger RD, Thompson JS, Shostrom VK, Carlson MA (2015). Effect of proximal versus distal 50% enterectomy on nutritional parameters in rats preconditioned with a high-fat diet or regular chow. Sci. Rep..

[32] Falken Y, Hellstrom PM, Holst JJ, Naslund E (2011). Changes in glucose homeostasis after Roux-en-Y gastric bypass surgery for obesity at day three, two months, and one year after surgery: role of gut peptides. J. Clin. Endocrinol. Metab..

[33] Kruit JK, Groen AK, van Berkel TJ, Kuipers F (2006). Emerging roles of the intestine in control of cholesterol metabolism. World J. Gastroenterol..

[34] Perna M, Baker M, Byrne TK, Morgan K (2011). Statins and the bariatric patient: characterization and perioperative effects of statin therapy in the gastric bypass patient. Am. Surg..

[35] Cavin JB (2016). Differences in alimentary glucose absorption and intestinal disposal of blood glucose after Roux-en-Y gastric bypass vs sleeve gastrectomy. Gastroenterology.

[36] Saeidi N (2012). Sleeve gastrectomy and Roux-en-Y gastric bypass exhibit differential effects on food preferences, nutrient absorption and energy expenditure in obese rats. Int. J. Obes. (Lond.).

[37] Brown RC, Kelleher J, Losowsky MS (1979). The effect of pectin on the structure and function of the rat small intestine. Br. J. Nutr..

[38] Brunham LR (2006). Intestinal ABCA1 directly contributes to HDL biogenesis in vivo. J. Clin. Invest..

[39] Daniel RS, Devi KS, Augusti KT, Sudhakaran Nair CR (2003). Mechanism of action of antiatherogenic and related effects of Ficus bengalensis Linn. flavonoids in experimental animals. Indian J. Exp. Biol..

[40] Kovanen PT, Nikkila EA, Miettinen TA (1975). Regulation of cholesterol synthesis and storage in fat cells. J. Lipid Res..

[41] Burstein M, Scholnick HR, Morfin R (1970). Rapid method for the isolation of lipoproteins from human serum by precipitation with polyanions. J. Lipid Res..

[42] Kanehisa M, Furumichi M, Tanabe M, Sato Y, Morishima K (2017). KEGG: new perspectives on genomes, pathways, diseases and drugs. Nucleic Acids Res..

