# Author Correction: “Dysfunctions” induced by Roux-en-Y gastric bypass surgery are concomitant with metabolic improvement independent of weight loss

**DOI:** 10.1038/s41421-020-0163-1

**Published:** 2020-04-28

**Authors:** Meiyi Li, Zhiyuan Liu, Bangguo Qian, Weixin Liu, Katsuhisa Horimoto, Jie Xia, Meilong Shi, Bing Wang, Huarong Zhou, Luonan Chen

**Affiliations:** 1 Key Laboratory of Systems Biology, Center for Excellence in Molecular Cell Science, Institute of Biochemistry and Cell Biology, Shanghai Institutes for Biological Sciences, Chinese Academy Sciences, Shanghai, 200031 China; 2 Institute of Fudan-Minhang Academic Health System, Minhang Hospital, Fudan University, Shanghai, 201199 China; 3 Molecular Profiling Research Center for Drug Discovery, National Institute of Advanced Industrial Science and Technology, Tokyo, Japan; 4 Shanghai Ninth People’s Hospital, Shanghai Jiao Tong University School of Medicine, Shanghai, 200011 China; 5 ShermanCollege of Chiropractic, Boiling Springs, SC 29316 USA; 6 Center for Excellence in Animal Evolution and Genetics, Chinese Academy of Sciences, Kunming, 650223 China; 7 School of Life Science and Technology, ShanghaiTech University, Shanghai, 200031 China; 8 Shanghai Research Center for Brain Science and Brain-Inspired Intelligence, Shanghai, 201210 China

**Keywords:** Mechanisms of disease, Transcriptomics

Correction to: *Cell Discovery* (2020) 6:4

10.1038/s41421-019-0138-2 Published online 28 January 2020

In the original publication of this article^1^, one of the funding was missing in the Acknowledgements part. The corrected Acknowledgements appear below:

**Acknowledgements**

This study was supported by National Key R&D Program of China (2017YFA0505500), the Strategic Priority Research Program of the Chinese Academy of Sciences (XDB13040700), National Natural Science Foundation of China (81471047, 31771476, 31930022 and 81802354), and Shanghai Sailing Program (18YF1420700). The authors are grateful to Dr. S. Anderson for English editing of the manuscript.
