# Supplementary material for: “Dysfunctions” induced by Roux-en-Y gastric bypass surgery are concomitant with metabolic improvement independent of weight loss
Source: Cell Discov. 2020 Jan 28;6:4. doi: 10.1038/s41421-019-0138-2 (PMC6985254; doi:10.1038/s41421-019-0138-2)
Supplement: Supplementary file 1 — Supplementary Material [file 41421_2019_138_MOESM1_ESM.pdf]

Supplementary Information

″Dysfunctions” induced by Roux-en-Y gastric bypass surgery are concomitant with metabolic improvement independent of weight loss

Supplementary Fig. 1 Molecular network within genes of four transition groups

Supplementary Fig. 2 Overview of metabolic pathways enriched by associated genes of four transition groups

Supplementary Fig. 3 Newly synthesized HDL-CHOL between STZ-S-30 and STZ rats

Supplementary Fig. 4: [^{14}C] glucose biodistribution

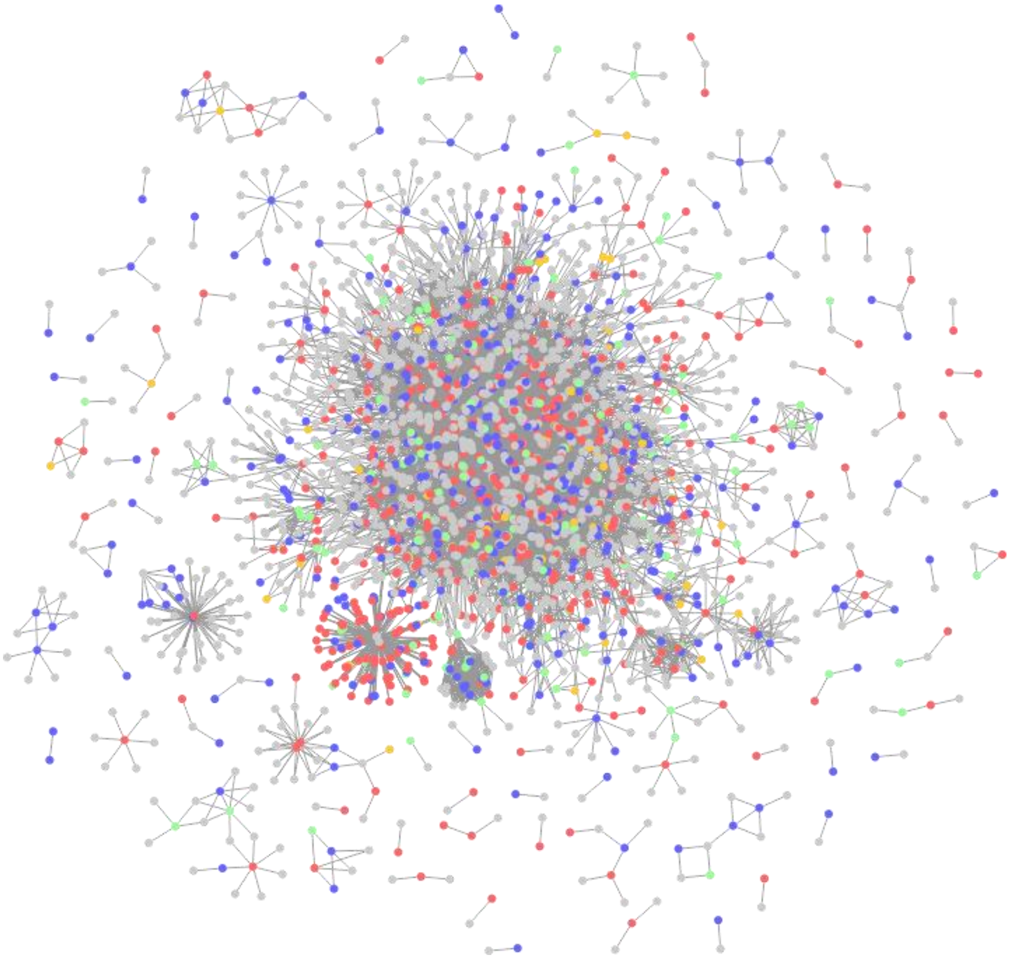

Supplementary Fig. 1. Molecular network within genes of four transition groups

To study coordination of the unchanged genes and other groups in terms of molecular interactions and systemic functions, we constructed subnets by integrating genes of each transition group according to knowledge-based molecular interactions in rats. Significance of colors (green, blue, red, yellow) as in Fig. 3.

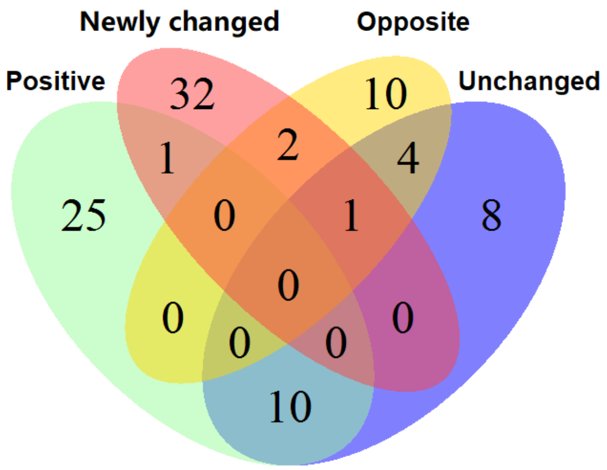

Supplementary Fig. 2. Overview of metabolic pathways enriched by associated genes of four transition groups

To study the functional coordination of four transition groups in terms of metabolic processes, we measured overlapping metabolic pathways not enriched by members of these transition groups.

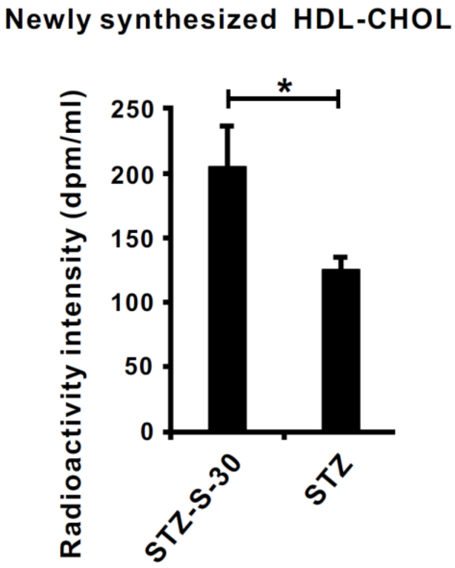

Supplementary Fig. 3. Newly synthesized HDL-CHOL between STZ-S-30 and STZ rats

To confirm that RYGB can induce increase of newly synthesized HDL-CHOL independent of total HDL-CHOL level, we measured newly synthesized plasma HDL-CHOL in STZ-S-30 and STZ groups through [^{14}C] tracer (HDL synthesized in intestine is secreted eventually into bloodstream), and observed significant increase of newly synthesized plasma HDL-CHOL in STZ-S-30.

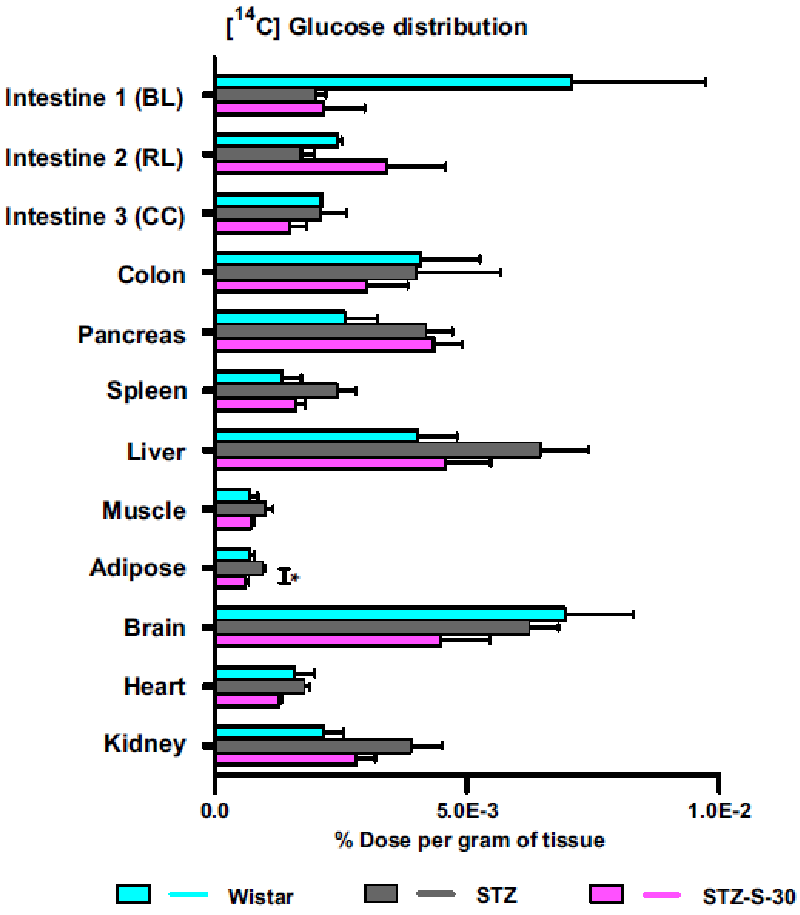

Supplementary Fig. 4. [^{14}C] glucose biodistribution

[^{14}C] Glucose was administered orally according to body weight. Three h later, [^{14}C] glucose biodistribution was measured in the three groups. For each tissue type, [^{14}C] glucose level did not differ significantly among the three groups.
